# Anticancer Efficacy of Polyphenols and Their Combinations

**DOI:** 10.3390/nu8090552

**Published:** 2016-09-09

**Authors:** Aleksandra Niedzwiecki, Mohd Waheed Roomi, Tatiana Kalinovsky, Matthias Rath

**Affiliations:** Dr. Rath Research Institute, 1260 Memorex Drive, Santa Clara, CA 95050, USA; w.roomi@drrath.com (M.W.R.); t.kalinovksy@drrath.com (T.K.); m.rath@drrath.com (M.R.)

**Keywords:** polyphenols, tumor growth, metastasis, Matrigel invasion

## Abstract

Polyphenols, found abundantly in plants, display many anticarcinogenic properties including their inhibitory effects on cancer cell proliferation, tumor growth, angiogenesis, metastasis, and inflammation as well as inducing apoptosis. In addition, they can modulate immune system response and protect normal cells against free radicals damage. Most investigations on anticancer mechanisms of polyphenols were conducted with individual compounds. However, several studies, including ours, have indicated that anti-cancer efficacy and scope of action can be further enhanced by combining them synergistically with chemically similar or different compounds. While most studies investigated the anti-cancer effects of combinations of two or three compounds, we used more comprehensive mixtures of specific polyphenols and mixtures of polyphenols with vitamins, amino acids and other micronutrients. The mixture containing quercetin, curcumin, green tea, cruciferex, and resveratrol (PB) demonstrated significant inhibition of the growth of Fanconi anemia head and neck squamous cell carcinoma and dose-dependent inhibition of cell proliferation, matrix metalloproteinase (MMP)-2 and -9 secretion, cell migration and invasion through Matrigel. PB was found effective in inhibition of fibrosarcoma HT-1080 and melanoma A2058 cell proliferation, MMP-2 and -9 expression, invasion through Matrigel and inducing apoptosis, important parameters for cancer prevention. A combination of polyphenols (quercetin and green tea extract) with vitamin C, amino acids and other micronutrients (EPQ) demonstrated significant suppression of ovarian cancer ES-2 xenograft tumor growth and suppression of ovarian tumor growth and lung metastasis from IP injection of ovarian cancer A-2780 cells. The EPQ mixture without quercetin (NM) also has shown potent anticancer activity in vivo and in vitro in a few dozen cancer cell lines by inhibiting tumor growth and metastasis, MMP-2 and -9 secretion, invasion, angiogenesis, and cell growth as well as induction of apoptosis. The presence of vitamin C, amino acids and other micronutrients could enhance inhibitory effect of epigallocatechin gallate (EGCG) on secretion of MMPs. In addition, enrichment of NM with quercetin (EPQ mix) enhanced anticancer activity of NM in vivo. In conclusion, polyphenols, especially in combination with other polyphenols or micronutrients, have been shown to be effective against multiple targets in cancer development and progression, and should be considered as safe and effective approaches in cancer prevention and therapy.

## 1. Introduction

Polyphenols comprise a diverse group of secondary metabolites abundant in plants, where they play key roles in regulating growth, metabolism, protecting against UV radiation and various pathogens. More than 8000 polyphenolic compounds have been identified in various plant species. Polyphenols have been subjected to numerous studies, investigating their potential health benefits, including protection against oxidative stress, cardiovascular disease, diabetes, asthma, neurodegenerative disease and even aging [1]. Particular interest in these naturally occurring plant components has been kindled by the search for new chemopreventive agents that are more effective and less toxic than conventional therapies. As such, this group of substances has been studied for anticarcinogenic properties, such as modulating cell proliferation, tumor growth, angiogenesis, metastasis, inflammation and apoptosis [2,3].

Polyphenols are classified based on the number of phenol rings and the structural elements that bind these rings to one another. The groups include: phenolic acids, stilbenes, lignans, and flavonoids. Flavonoids, which have both antioxidant and anti-inflammatory properties, are found in fruits, vegetables, legumes, red wine, and green tea. They are subdivided into six classes: flavonols, flavones, isoflavones, flavanones, anthocyanidins, and flavanols (catechins and proanthocyanidins) [4]. Flavonols, the most ubiquitous flavonoids in foods, are generally present at relatively low concentrations. Quercetin and kaempferol are the main representatives, and their richest sources are onions, curly kale, leeks, broccoli, and blueberries [4].

Flavones, consisting mainly of glycosides of luteolin and apigenin, are less common than flavonols in fruit and vegetables; parsley and celery are the main edible sources of flavones [4]. Flavanones, present in tomatoes and certain aromatic plants such as mint, are present in high concentrations only in citrus fruit. Isoflavones, flavonoids with structural similarities to estrogens, have the ability to bind to estrogen receptors, and thus are classified as phytoestrogens. They are found almost exclusively in leguminous plants. Flavanols occur as catechins (a monomer form) and proanthocyanidins (the polymer form).

Catechins are found in many types of fruit, red wine, green tea and chocolate. Gallocatechin, epigallocatechin, and epigallocatechin gallate (EGCG) are found in teas, seeds of leguminous plants and grapes [5,6]. Green tea, a rich source, contains up to 200 mg catechins in a cup of tea [7]. Flavanols are not glycosylated in foods as are other classes of flavonoids; thus tea epicatechins remain very stable when exposed to heat under acidic pH. Only 15% of these substances are degraded after 7 h in boiling water at pH 5 [8].

Among phenolic acids, hydroxybenzoic acids are found in tea and more common hydroxycinnamic acids are found in cinnamon, coffee, blueberries, kiwis, plums, apples, and cherries [4]. These acids are mostly found as glycosylated derivatives of esters of quinic acid, shikimic acid and tartaric acid [4]. Ferrulic acid is the most abundant phenolic acid found in cereal grains and in wheat; it may represent up to about 90% polyphenols [9].

Stilbenes are found in low quantities in our diet, which may be not sufficient to exercise significant health effects; larger quantities can be provided in concentrated extracts or in the form of purified compounds. Resveratrol is a key stilbene, found especially in red wine and peanuts, which has been extensively studied for its anticarcinogenic and other health effects [10].

Lignans are found in flax seeds, legumes, cereals, grains, fruits, algae, and certain vegetables [4]. Their concentration in linseed is about 1000 times as high as in other food sources [11]. Plant lignin, secoisolariciresinol diglycoside (SDG) and its metabolites have shown promise in reducing carcinogenic tumors, in particular hormone-sensitive ones such as breast, endometrium and prostate tumors [12].

## 2. Aspects Associated with Bioavailability of Polyphenols

Bioavailability defines the amount of nutrient that is digested, absorbed and metabolized in normal biochemical pathways. Most polyphenols are present in food in the form of esters, glycosides or polymers, and are metabolized by a common pathway. Before absorption, most polyphenols have to be hydrolyzed by intestinal enzymes or microflora present in colon. Only aglycones can be absorbed via the small intestine [13]. Subsequently, during absorption, polyphenols undergo extensive modification by conjugation in the intestinal cells and further processing in the liver through methylation, sulfation and glucuronidation [13]. Therefore, their forms detected in the blood and tissues differ from the ones present in food. In addition, the most biologically active polyphenols are not necessarily the most common ones in the diet.

Metabolic activity of polyphenols is dependent on intrinsic activity, relative absorption from the intestine, rate of metabolism and of elimination. In addition, digestive or hepatic metabolic activity may render the metabolites that reach the blood and target organs to differ in biological activity from their native form. Polyphenol metabolites circulate in the blood bound to proteins, mainly albumin, with binding affinity largely defined by their structural properties [14]. Protein binding can affect the rate of their delivery to cells and tissues.

Polyphenols are able to penetrate tissues, in particular the intestine and liver, where they are metabolized. Their excretion occurs through urine (mainly small conjugates) and more extensively conjugated metabolites are secreted in bile. It appears that the amount of metabolite in urine roughly corresponds to its maximum concentration in plasma. It is quite high for citrus fruit flavanones and decreases from isoflavones to flavonols. Thus, understanding polyphenol bioavailability is essential for determining their health effects.

## 3. Antitumor Effects of Select Polyphenols

Development of cancer is a multi-stage process that involves initiation, promotion and progression. Dietary polyphenols can affect and modulate multiple diverse biochemical processes and pathways involved in carcinogenesis [15]. In addition, they can act as biological response modifiers supporting immune system function, as well as protecting living cells against damage from free radicals. Although polyphenols in fruits and vegetables are widely implicated in cancer prevention, few protective effects of individual compounds have been firmly confirmed in clinical trials due to differences in dosing, timing and other confounding factors.

Several cancer preventive mechanisms have been identified as affected by polyphenols, including prevention of oxidation, detoxification of xenobiotics, induction of apoptosis, as well as estrogenic/anti-estrogenic activity, stimulating effects on immune system function, anti-inflammatory properties and their effects on the cellular signaling system. Among these are effects on nuclear factors, such as NF-κB or activator protein 1 (AP-1), which play central roles in cellular signaling cascades, regulating DNA transcription, gene expression in response to different stimuli, cell proliferation and survival [16,17]. Most investigations have been conducted with individual polyphenols in order to increase our understanding of biological and cellular mechanisms of their anticancer efficacy. However, final effects of these compounds are largely influenced by their interactions with other natural components, both at the cellular and organ levels. These aspects have been rarely evaluated and studied. Below we will discuss key studies investigating anti-cancer effects of individual polyphenols, as well as their efficacy when used in various combinations, the approach which has been promoted in our research.

### 3.1. Curcumin

This active ingredient of a rhizome of turmeric has been known for its anti-oxidant properties and many health benefits. It has been demonstrated in different cancer models that curcumin can inhibit cellular proliferation and angiogenesis, block cell cycle progression in tumor cells, and induce apoptosis [18,19,20]. Aoki et al. demonstrated inhibition of glioma cell xenograft tumor growth by curcumin [21]. Studies by Anand indicate that in addition to modulating cancer cell proliferation, angiogenesis and inducing apoptosis, curcumin can affect cancer invasion, and metastasis [22]. The anti-cancer efficacy of curcumin may involve a variety of mechanisms. Study of pancreatic cancer in nude mice has shown that curcumin can suppress pancreatic cancer cell proliferation and angiogenesis by inhibiting NF-ĸB-regulated gene products such as cyclin D1, cmyc, Bcl-2, Bcl-xL, and apoptosis protein-1, COX-2, MMP, and vascular endothelial growth factor (VEGF) [18]. Various studies, including the use of lung models, point to curcumin affecting the mechanisms involving inhibition of the signal transducer and activation of transcription Stat3 pathways [23], as well as matrix metalloproteinases and VEGF [18].

Curcumin’s anti-tumor effects may also be due to its interactions with arachidonate metabolism and in vivo anti-angiogenic properties [24]. Some studies indicate curcumin interacts with vitamin D receptors, which would explain its preventive properties against colon cancer where vitamin D has strong anti-cancer effects [25]. Curcumin also appears to work in synergy with isoflavones in suppressing prostate specific antigen (PSA) production in prostate cells [26]. Gupta et al. have reviewed clinical trial research on curcumin and have concluded that curcumin, either alone or in combination with other agents, has demonstrated potential against various cancers, including colorectal, pancreatic, breast, prostate, lung, multiple myeloma, and head and neck squamous cell carcinoma [27]. Overall, chemoprevention and anticancer effects of curcumin are multimodal and complex. Molecular pathways and targets modulated by curcumin have been discussed in details in other review articles [28,29].

### 3.2. Quercetin

Cancer preventive effects of quercetin include induction of cell cycle arrest, apoptosis and antioxidant functions [30]. Induction of apoptosis by quercetin in cancer cells during different cell cycle stages without affecting normal cells has been documented in various cancers in vivo and in vitro [30]. Quercetin has been reported to reduce both the risk and progression of cancer through their free radical scavenging activity [31,32]. It protects cells from oxidative stress, inflammation, and DNA damage due to its antioxidant properties and modulates growth of many cancer cell lines by blocking cell cycle progression and tumor cell proliferation and by inducing apoptosis [33,34,35,36]. In addition, quercetin provided protection against liver cancer development in rats injected with a cancer inducer [37]. Furthermore, injection of quercetin directly into breast tumors led to a significant reduction of their mass [38]. Epidemiological studies report that intake of quercetin-rich food reduced the risk of gastric cancer by 43% [31] and colon cancer by 32% [39]. Consumption of quercetin was also reported to reduce lung cancer risk by 51% and even in heavy smokers by 65% [32]. Intravenous administration of quercetin in patients with different types of cancer demonstrated decreased activity of the enzyme tyrosine kinase, an enzyme required for tumor growth, in nine of 11 patients [40]. There is a growing interest among scientists in exploring synergistic interactions of quercetin with standard chemotherapeutics. Both in vitro and in vivo studies have shown that quercetin can potentiate the efficacy of concomitant drugs by enhancing their bioavailability and accumulation, and by sensitizing cancer cells to these chemotherapeutics [41]. From a clinical perspective, this would allow dose reduction of toxic drugs, thereby alleviating their severe side effects.

### 3.3. Resveratrol

Resveratrol has demonstrated to be effective in preventing all stages of cancer development and has been found effective in most cancers including prostate, breast, stomach, colon, lung, thyroid and pancreatic cancer cell lines [42]. Bishayee et al. reported that resveratrol can affect carcinogenesis by modulating signal transduction pathways that control cell division and growth, apoptosis, inflammation, angiogenesis and metastasis [43]. In vivo, resveratrol has shown efficacy in preventing and treating skin, esophageal, intestinal, and colon tumors [44]. Various anti-cancer cellular mechanisms of resveratrol have been suggested, including inhibition of angiogenesis, metastasis and induction of apoptosis [38]. Resveratrol has been studied in patients with colon cancer to evaluate the pharmacokinetic and metabolite profiles in these patients [45]. In addition, resveratrol studied in healthy volunteers demonstrated that it is well tolerated and modulates enzyme systems involved in carcinogen activation and detoxification [46]. Resveratrol has promising anti-cancer potential but, because of its poor bioavailability, the best efficacy has been limited to tumors it can come into direct contact with (e.g., skin cancers or gastrointestinal tract cancers).

### 3.4. Cruciferous Plant Extracts

Cruciferous vegetables of the *Brassica* genus, such as broccoli, cabbage, cauliflower and others contain a number of nutrients and phytochemicals with cancer preventive properties, including carotenoids, chlorophyll and fibers. Their unique anti-cancer characteristic relates to being a source of large quantities of sulfur containing natural compounds known as glucosinolates [47]. After hydrolysis they form bioactive isothiocyanates and indole-3-carbinol [48,49]. By 2011 more than 132 glucosinolates with their unique hydrolysis products had been identified in plants [50]. Epidemiological evidence suggests that high intake of cruciferous vegetables can lower risk of some cancers, including colon and lung cancers [48]. Numerous in vitro and in vivo studies suggest that dietary compounds from cruciferous plants may have potent chemopreventive properties by acting through different molecular mechanisms [51,52,53,54,55,56]. This includes many studies, which have shown that cruciferous vegetables can help prevent the onset and inhibit the progression of colon, breast, prostate, thyroid, cervical, and other cancers [57,58,59,60,61,62,63,64,65,66]. It has been shown that cruciferous bioactive components can facilitate detoxification and excretion of carcinogens, protect against oxidative stress, inhibit cancer cell proliferation and increase apoptosis, resulting in inhibition of tumor growth [67].

### 3.5. Green Tea

Green tea extract, rich in catechins, has been subjected to numerous studies and shown to modulate cancer cell growth, metastasis, angiogenesis, and other aspects of cancer progression by affecting different mechanisms [68,69,70,71,72,73]. The four principal tea catechins are epicatechin (EC), epicatechin-3-gallate (ECG), epigallocatechin (EGC) and epigallocatechin-3-gallate (EGCG) [74]. It has been shown that three of these catechins, EGCG, EGC and ECG, have anticancer preventive activity, and that their combination with an inactive EC has synergistic effect on prostate cancer cells PC-9, by inducing apoptosis and inhibiting proliferation [75].

The most studied of all green tea catechins in relation to cancer is EGCG, which is found at the highest concentration in green tea (10%–15%) [75]. Gupta et al. demonstrated that EGCG potently induced apoptosis and suppressed cell growth in vitro by modulating expression of cell cycle regulatory proteins, activating killer caspases, and suppressing activation of NF-κB [76]. Harakeh et al. demonstrated the anti-proliferative and pro-apoptotic effects of EGCG in HTLV-1-positive and -negative cells. These effects included down-regulation of TGF-alpha, up-regulation of TGF-beta2, increased cell distribution in pre-G(1) phase and upregulation of p53, Bax and p21 proteins expression while down-regulating Bcl-2alpha [73]. EGCG was reported to control and promote IL-23 dependent DNA repair, enhance cytotoxic T-cell activities and block cancer development by inhibiting carcinogenic signal transduction pathways [77]. EGCG was also shown to modulate several biological pathways, including growth factor-mediated pathway, the mitogen activated protein kinase-dependent pathway, and ubiquitin/proteasome degradation pathways [78]. Clinically, in a study of over 8000 individuals, daily consumption of green tea demonstrated delayed cancer onset; furthermore, breast cancer patients experienced a lower recurrence rate and longer remission [75]. Another clinical study found that oral ingestion of EGCG (200 mg per os) for 12 weeks was effective in patients with human papilloma virus-infected cervical lesions [79].

While EGCG is the most studied catechin in cancer, the in vitro study in human pancreatic ductal adenocarcinoma (PDAC) cells showed that two minor green tea catechins, ECG and EG, had much stronger anti proliferative and anti-inflammatory effects, including inhibition of NF-κB, IL8 and uPA, than EGCG [80].

Epigallocatechin gallate (EGCG) has poor bioavailability in rats and humans due to oxidation, metabolism and its efflux. In addition, specific interactions with other polyphenolic compounds can modulate its metabolic efficacy. However, a study by Kale et al. [81] showed that green tea extract consumed together with onion, which is rich in quercetin, can significantly enhance bioavailability of EGCG in humans. Wang et al. confirmed this effect, showing that quercetin affects EGCG by decreasing its methylation [82]. EGCG-quercetin interactions resulted in enhanced anti-proliferative effects of EGCG in androgen-dependent and androgen-independent prostate cancer cells [83]. EGCG bioavailability is affected by numerous compounds as its consumption with cereal and milk resulted in lower EGCG blood levels [84]. This indicates that proper selection of compounds and their combinations are important in achieving the desired biological effect.

## 4. Benefits of Nutrient Combinations—Pleiotropic Effects

Numerous in vitro and in vivo studies provide support for use of polyphenols in cancer prevention by applying them in combinations with other micronutrients for achieving pleiotropic effects. As mentioned previously, one of major problems with using polyphenols as anticancer agents individually is their poor bioavailability in the human body. In addition, their interactions with other natural compounds in a diet may hinder or complicate consistency of their efficacy [85]. Therefore, specifically designed combinations of several polyphenols or combinations of polyphenols with other natural agents aimed at defined biological targets will expand metabolic effects of constituents of such mixtures in controlled and reproducible ways. In addition, proper combinations of micronutrients enable use of lower doses of individual components without compromising their efficacy, rather than expanding the scope of cellular mechanisms affected. This novel approach opens up a possibility of developing more effective strategies against various human pathologies, including cancer.

Several in vitro and in vivo studies have shown that combinations of two or three polyphenols were more effective in inhibiting cancer growth than treatment with a single compound. Our research has demonstrated that multi-nutrient combinations, through their reciprocal interactions, including synergy, can modulate bioavailability of natural compounds and exercise pleiotropic effects by affecting multiple metabolic pathways involved in carcinogenesis simultaneously. Below we present a few examples of specific and effective anti-cancer nutrient mixtures. They include a combination of different polyphenols and combinations of polyphenols with vitamins and other micronutrients, which were selected to specifically target defined key cellular mechanisms involved in carcinogenesis.

### 4.1. Anticancer Effects of a Combination of Different Polyphenols

Several studies investigated the efficacy of mixtures containing two or three different polyphenols. These include combinations of EGCG with quercetin in prostate cancer cells [86,87], and EGCG with resveratrol in prostate cancer cell lines [88] and breast cancer MCF-7 cell line [89]. Combinations studied also included curcumin with EGCG and EC in lung cancer cell lines [90,91], curcumin with EGCG in human B-cell chronic leukemia [92] and in xenograft mouse models implanted with MDA-MB-231 breast cancer cells [93] and lung A549 cells [91]. Piao et al. [94] demonstrated anti-cancer effects of a tri-nutrient combination composed of curcumin, resveratrol and epicatechin gallate on cell viability, clonogenic survival, apoptosis and tumor growth in HPV-positive head and neck squamous cell carcinoma.

These findings indicate that more comprehensive mixtures of nutrients with similar or complementary anti-cancer mechanisms would allow for enhanced complementary or synergistic interactions between these compounds, thereby expanding different cancer mechanisms simultaneously and in a controlled fashion. Therefore, we combined the compounds previously investigated in combinations and cruciferous extract in order to enhance and expand their anti-cancer mechanisms through additive and synergistic effects. This mixture of nutrients (PB) included: quercetin, 400 mg; Cruciferex™ (prioprietary extract from cabbage, cauliflower, broccoli and carrots), 400 mg; turmeric root extract containing 95% curcuminoids, 300 mg; resveratrol, 50 mg; and standardized green tea extract (80% polyphenols), 300 mg. See Table 1 for summary of PB studies using in vitro and in vivo approaches. These included human Fanconi anemia head and neck squamous cell carcinoma (HNSCC), which with acute myeloid leukemia, are the major causes of mortality and morbidity in Fanconi anemia patients. We found that nude mice injected with OHSU-974 cells and subsequently fed a diet supplemented with 1% PB demonstrated inhibition of tumor growth by 67.6% (*p* < 0.0001) and tumor burden by 63.6% (*p* < 0.0001) [95]. In vitro study demonstrated dose-dependent inhibition of OSHU-974 cell proliferation, with cell toxicity detected at 27% (*p* = 0.0003) and 48% (*p* = 0.0004) at PB concentrations of 75 and 100 µg/mL, respectively [95]. These cells constitutively secrete matrix metalloproteinase (MMP)-2 and, after stimulation by phorbyl 12-myristate (PMA), also MMP-9. The secretion of MMPs by both stimulated and unstimulated cells was suppressed by PB in a dose-dependent manner, with total block of both at 50 µg/mL, which is below PB’s toxicity level [95]. This concentration was also effective in inhibiting cancer cell migration (by scratch test) and invasion through Matrigel, apparently not related to cell toxicity, as no visible morphological changes were detected at this and lower concentrations [95].

The inhibitory effects of PB were also observed in fibrosarcoma, which is an aggressive and highly metastatic cancer of connective tissue, and in melanoma, which causes the majority of skin cancer-related deaths, secondary to metastasis. The in vitro studies used human fibrosarcoma cell line HT-1080 [96] and melanoma A2058 cells [97]. The study showed that PB inhibited cell proliferation of human fibrosarcoma cells by 60% at 25 μg/mL and 80% at 50 μg/mL, and inhibited both MMP-2 and -9 secretion in a dose-dependent manner, with total inhibition at 50 μg/mL [96]. At PB concentration of 25 μg/mL, invasion of HT-1080 cells through Matrigel was totally blocked [96]. Melanoma cells were more sensitive to PB, as their proliferation was inhibited by 80% at 25 ug/mL and MMP-2 and -9 secretion and Matrigel cell invasion blocked completely at 50 μg/mL, indicating strong anti-invasive properties of this polyphenol mixture [97]. PB also induced cell apoptosis in both cell lines in a dose-dependent fashion.

### 4.2. Anticancer Effects of Combinations of Polyphenols, with Vitamins and Other Compounds

Combinations of polyphenols with mixtures of vitamins, amino acids and other micronutrients are rarely utilized in cancer research. We initiated this multi-targeted approach over a decade ago, demonstrating that a combination of EGCG with specific micronutrients showed anti-proliferative and anti-invasive effects in human breast (MDA-MB-231), colon (HCT 116) and melanoma (A-2058) cancer cells [98]. The investigated combination included EGCG from green tea together with ascorbic acid, lysine, proline, arginine, *N*-acetyl cysteine, selenium, copper and manganese. This mixture, designated as NM, was tested in vitro and in vivo against a few dozen human cancer cell lines. The composition of this specific micronutrient mixture exemplifies the importance of extracellular matrix-mediated processes in developing a general strategy of effective control of cancer. While most of natural compound combinations target intracellular regulatory mechanisms of tumorigenesis, our approach expands it to include connective tissue integrity as a common element involved in tumor growth, invasion and metastasis. The role of this natural anti-cancer barrier is frequently overlooked in therapeutic approaches to cancer. However, all critical metabolic steps in cancer require degradation of basement membrane and extracellular matrix components as the physical barrier restraining growth, migration and invasion of tumor cells. Increased activity of proteinases, such as MMPs and plasmin, accompanied by down-regulation of their natural inhibitors has been correlated with aggressiveness of cancer and angiogenesis, associated with neovascularization of tumors [99,100]. This new approach to curbing cancer growth by strengthening the connective tissue in the body was introduced by Rath in the 1990s [101]. This can be achieved with micronutrients essential for the synthesis and structure of connective tissue (vitamin C, lysine, proline, and copper manganese) and inhibitors of enzymatic breakdown of collagen facilitated by malignant cells (i.e., lysine). In addition to its essential role in collagen production, vitamin C has shown to be an effective anti-cancer agent (pro-oxidant effects) [102,103] and in cancer prevention (antioxidant effects) [104]. The presence of polyphenols in this mixture, in addition to selenium, *N*-acetylcysteine and arginine allows for expanding biological targets and enhancing several cellular mechanisms important in controlling cancer, such as immune system function. The mixture was formulated in two variants in regard to its polyphenol components: including both EGCG and quercetin (EPQ) and containing EGCG (NM) as the sole polyphenol. Both compositions were tested against several human and murine cancer cell lines both in vivo and in vitro.

#### 4.2.1. EGCG in Combination with Vitamin C, Amino Acids and Other Micronutrients

The multiple aspects of anticancer efficacy of the combination of green tea extract rich in EGCG with ascorbic acid, lysine, proline, arginine, *N*-acetylcysteine, selenium, copper and manganese (NM) on several cancer cell lines in vivo and in vitro were presented in a review article [105].

This unique mixture demonstrated potent, significant suppression of hepatic and pulmonary metastasis in a murine model resulting in significant reduction in tumor size and tumor burden in all human cancer cell lines tested. In vitro studies demonstrated that NM was very effective in inhibition of cell proliferation (by MTT assay), MMP secretion (by gelatinase zymography), cell invasion (through Matrigel), cell migration (by scratch test), angiogenesis (VEGF and other angiogenic models), induction of apoptosis (by live green caspase) and induction of pro-apoptotic genes in many diverse cancer cell lines. A more recent study conducted with the NM mixture illustrates its efficacy against multiple parameters of cancer growth and progression in vivo and in vitro. We used an orthotopic breast cancer model to evaluate the development of tumors and metastasis, challenging mice with breast cancer 4T1 cells into the mammary pad, and also studied in vitro activity of NM on cell proliferation, MMP secretion, cell migration and invasion [106]. See Table 2 for summary of results. In contrast to human tumor cell models, murine tumor cell models often metastasize more effectively and display metastatic characteristics more similar to those observed in cancer patients. The 4T1 mammary carcinoma model was chosen since the primary tumor grows in the anatomically correct site and, as in human breast cancer, 4T1 metastatic disease develops spontaneously from the primary tumor. In addition, metastatic spread of 4T1 metastases to other organs and the draining lymph nodes is similar to that of human mammary cancer. The results showed that NM inhibited tumor weight and burden by 50% (*p* = 0.02) and 53.4% (*p* < 0.0001), respectively, and metastasis, by reducing mean number of colonies by 87% (*p* < 0.0001) and mean weight of lungs by 60% (*p* = 0.0001) compared to Control mice [106]. Metastasis to liver, spleen, kidney and heart was significantly reduced with NM supplementation [106].

#### 4.2.2. Inhibitory Effects of EGCG Applied Individually, and in Combination with Other Micronutrients on MMPs

In a previous study, we compared the anticancer effects of EGCG alone, in green tea and in combinations with micronutrients on the critical aspect of malignancy. Since type IV collagenase matrix metalloproteinases, especially MMP-2 and MMP-9, have been found to promote invasion and metastasis of malignant tumors, we investigated the relative inhibitory effects in MMP-2 and -9 by EGCG, the main polyphenol in green tea extract (GTE) to that of GTE and to that of NM as a whole using four cancer cell lines expressing MMP-2, MMP-9 or both, to determine if there is an additive or synergistic effect from combinations of these agents [107]. The cell lines tested included human fibrosarcoma (HT-1080), hepatocellular carcinoma (SK-Hep-1), glioblastoma (T-98G) and uterine leiomyosarcoma (SK-UT-1). The cells were also treated with PMA 100 ng/mL to study enhanced expression of MMP-9. Secretion of MMPs was assessed by gelatinase zymography. Fibrosarcoma and hepatocellular carcinoma cells expressed both MMP-2 and MMP-9, glioblastoma cells MMP-2 and PMA-induced MMP-9, and uterine leimyosarcoma cells only PMA-induced MMP-9. NM was the most potent dose-dependent inhibitor of MMPs, followed by green tea extract and EGCG. See Table 3 for summary of results. These results suggest the enhanced efficacy of nutrients working in synergy to modulate complex pathways such as MMP expression.

#### 4.2.3. Quercetin in Enhancing the Micronutrient Mixture Efficacy in Established Breast Cancer Tumors

We observed that including quercetin in the nutrient mixture containing EGCG has beneficial effects when administered to rats at early stages of breast tumor growth. Breast tumors were induced by IP administration of *N*-methyl-*N*-nitrosourea to Wistar rats [108]. After tumors were evident in all rats, the animals were divided into groups and fed one of the following regimens for 60 days: control diet; 30 mg green tea extract alone; 30 mg green tea extract in combination with NM; or a diet containing 30 mg of green tea extract, NM and quercetin. The results showed that tumor size/rat was significantly lower in all supplemented groups than in the Control group. The rats on the diet containing NM and quercetin showed significantly lower values for tumor incidence, mean tumor volume and mean tumor weight compared to the Control, the green tea extract and the NM groups [108]. Rats in the Control group developed 24 carcinomas (mostly of grade III severity), contrasted with six carcinomas (all of which were of grade II severity) in the NM supplemented group containing quercetin. The incidence, mean tumor volume and mean tumor weight of the green tea extract group alone (2.0, 7.27 cc, and 5.33 g, respectively) were higher than those of the NM group (1.4, 3.39 cc, and 1.71 g, respectively), and that was greater than that of the quercetin + NM group (1.0, 2.69 cc, and 1.35 g, respectively); however, the differences did not reach statistical significance. Higher anti-cancer efficacy of the mixture in the presence of quercetin might be related to its effects on EGCG bioavailability, as the results showed that administration of EGCG in the mixture with vitamins, amino acids and trace element (NM) resulted in a modest increase of its plasma concentration to 45.81 ng/mL from 35.84 ng/mL with EGCG administered alone. The enrichment of this mixture with quercetin resulted in increased plasma EGCG level to 65.94 ng/mL.

#### 4.2.4. Anticancer Effects of EGCG plus Quercetin in the Micronutrient Mixture

The studies presented below illustrate the anti-cancer potential of EGCG and quercetin in combination with the micronutrient mixture against ovarian cancer using two different cell lines (ES-2 and A-2780) [109,110]. See Table 4 for summary of results. EPQ contains the following nutrients in the relative amounts indicated: vitamin C (as ascorbic acid and as Mg, Ca ascorbates, and ascorbyl palmitate) 700 mg; l-lysine 1000 mg; l-proline 750 mg; l-arginine 500 mg; *N*-acetyl cysteine 200 mg; standardized green tea extract (80% polyphenol) 1000 mg; quercetin as quercetin dihydrate, from *Saphora japonica* 50 mg; selenium 30 μg; copper 2 mg; manganese 1 mg.

**Figure 1:**
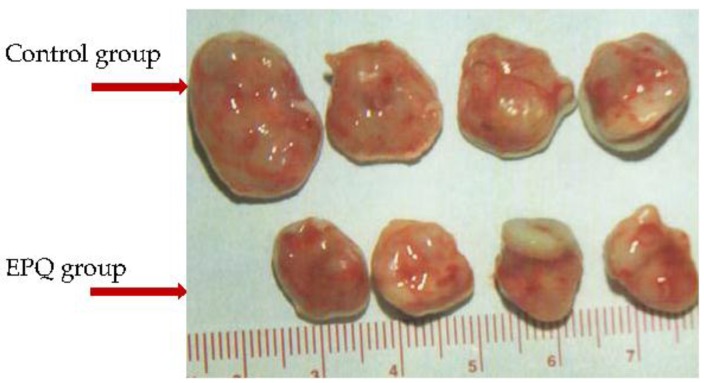
Gross photographs of representative tumors from Control and EPQ groups of mice inoculated with ovarian cancer ES-2 cells. Mean tumor weight and burden of EPQ group tumors were inhibited by 59.2% (*p* < 0.0001) and 59.7% (*p* < 0.0001) compared to Control group, respectively.

Epithelial ovarian carcinoma is the leading cause of death from gynecological malignancy due to metastasis and recurrence. Our previous publication on athymic female mice inoculated with ES-2 ovarian cancer cells showed that dietary intake of EPQ (0.5%, w/w) inhibited weight and burden of tumors by 59.2% (*p* < 0.0001) and 59.7% (*p* < 0.0001), respectively [109] (see Figure 1). A subsequent study on ovarian carcinoma used IP injection of ovarian cancer A-2780 cells into athymic female mice and focused on ovarian tumor growth and lung metastasis [110]. In the A-2780 study, all Control mice developed large ovarian tumors, whereas five out of six mice in the EPQ group developed no tumors, and one, only a small tumor [110]. EPQ suppressed tumor growth by 87% (*p* < 0.0001). In addition, all animals in the Control group had lung metastasis while, in contrast, no metastasis was observed in the EPQ group of mice [110]. In vitro, the effects of EPQ on cell proliferation, MMP secretion, invasion through Matrigel, migration by scratch test and morphology were evaluated in ES-2 [109] and A2780 [110] cells. EPQ exhibited 35% toxicity over the control in ES-2 cells [109] and 80% in A-2780 cells [110] at 1000 µg/mL concentration. ES-2 cells demonstrated only MMP-2, with and without PMA, which was inhibited by EPQ in a dose dependent fashion, with near total inhibition at 1000 µg/mL [109]. A-2780 cells demonstrated only MMP-9 expression, which EPQ inhibited in a dose dependent fashion, with virtual total block at 250 μg/mL concentration [110]. Migration of ES-2 cells by scratch test and invasion through Matrigel were inhibited in a dose dependent manner with total block of invasion and migration at 500 µg/mL [109]. Invasion through Matrigel of A-2780 cells was completely inhibited by EPQ at 250 µg/mL [110]. H&E staining showed no morphological changes below 500 μg/mL EPQ in A-2780 cells [110] and below 1000 μg/mL EPQ in ES-2 cells [109]. See Table 4 for summary of results.

An earlier study investigated the efficacy of a combination of polyphenols, quercetin and green tea, on prostate cancer xenograft tumor growth. The results confirmed the synergistic effects of this nutrient combination resulting in tumor growth inhibition by 45%, compared to 15% with quercetin and 21% with green tea only [111]. Although the experimental design and tumor type differed in the Wang studies from our research, both investigations point out to enhance anticancer effects of various nutrient combinations and the importance of connective tissue in confining tumor growth and metastasis.

## 5. Conclusions

Current cancer therapies based on chemotherapy and radiation are associated with significant side effects. Therefore, there is an urgent need for developing alternate or adjuvant approaches. Polyphenolic compounds, which are abundant from dietary sources, show great promise in cancer treatment, especially considering their safe use. Due to their ability to modulate multiple biological mechanisms involved in cancer initiation and progression, they offer more comprehensive therapeutic effects than single drugs. Bioavailability, as well as curative and preventive properties of these nutrients, can be enhanced and expanded in combination therapies that include natural compounds of the same or different chemical class. Combinations of polyphenols with micronutrients essential for maintaining integrity and stability of extracellular matrix offer expanded anti-cancer benefits. They include targeting complementary metabolic pathways important in curtailing cancer invasion and metastasis. Therefore, future research directions should expand to using natural compounds, especially in combinations, as safe, effective and affordable therapeutic approaches to cancer.

## Figures and Tables

**Table 1:** Anticancer effect of polyphenol mixture containing quercetin, curcumin, green tea, Cruciferex™, and resveratrol (PB) on cancer cell lines.

Cancer Cell Line and in Vivo Design	Tumor Growth Inhibition	In Vitro Inhibition
FA HNSCC Athymic male nude mice injected SQ with 3 × 10^6^ OHSU-974 cells Control group fed regular murine diet and PB group diet supplemented with PB 1% × 4 weeks [95]	Tumor weight by 67.6% (*p* < 0.0001) Tumor burden by 63.6%	Cell proliferation inhibited by 48% at 100 µg/mL PB; MMP-2 and -9 completely blocked at 50 µg/mL PB; cell migration and Matrigel invasion blocked at 50 µg/mL PB
Fibrosarcoma HT-1080 [96]	N/A	HT-1080 cell proliferation inhibited by 80% at 50 µg/mL PB; MMP-2 and -9 completely blocked at 50 µg/mL PB; Matrigel invasion blocked at 25 µg/mL PB; induction of dose-dependent apoptosis
Melanoma A-2058 [97]	N/A	A-2058 cell proliferation inhibited by 80% at 25 µg/mL PB; MMP-2 and -9 completely blocked at 50 µg/mL PB; Matrigel invasion blocked at 50 µg/mL PB; induction of dose-dependent apoptosis

**Table 2:** Anticancer effect of the combination of green tea extract with ascorbic acid, lysine, proline, arginine, *N*-acetyl cysteine, selenium, copper and manganese (NM) on breast 4T1 tumor [106].

Tumor Cell Line/In Vivo Design	Tumor Growth and Metastasis	In Vitro Results
Orthotopic injection of 5 × 10^5^ breast cancer 4T1 cells into the mammary pad of Balb C mice Control group fed regular murine diet and NM group diet supplemented with NM 0.5% × 4 weeks	Tumor weight reduced by 50% (*p* = 0.02) and tumor burden by 53.4% (*p* < 0.0001) in NM mice compared to Control mice Lung metastasis inhibited by 87% (*p* < 0.0001) in NM mice compared to Control mice Mean weight of lungs reduced by 60% (*p* = 0.0001) Metastasis to liver, spleen, kidney and heart significantly reduced in NM group compared to Control	Cell proliferation reduced by 50% at 250 µg/mL NM MMP-2 and -9 completely blocked at 1000 µg/mL NM Cell migration and Matrigel invasion blocked at 250 µg/mL NM

**Table 3:** Comparative cumulative expression of MMP-2 and -9 in cell lines treated with epigallocatechin gallate (EGCG), green tea extract (GTE) and the nutrient mixture (NM) [107].

	EGCG	GTE	NM	EGCG + PMA	GTE + PMA	NM + PMA
Fibrosarcoma HT-1080						
MMP-2	7.88	7.47	3.29	0.21	0.20	0
MMP-9	5.74	3.02	1.58	209.06	139.84	93.54
Hepatocellular carcinoma Sk-Hep-1						
MMP-2	1.21	1.10	0	0.77	0.55	0.29
MMP-9	256.51	187.28	26.59	611.90	593.80	508.28
Glioblastoma T-98G						
MMP-2	109.97	86.63	65.84	178.16	140.09	53.20
MMP-9	0.37	0.37	0.10	92.69	82.67	52.50
Uterine leimyosarcoma SK-UT-1						
MMP-2	0	0	0	51.36	49.97	34.30
MMP-9	0	0	0	87.42	87.30	77.95

**Table 4:** Anticancer effect of mixture containing polyphenols quercetin and green tea extract with ascorbic acid, lysine, proline, arginine, *N*-acetyl cysteine, selenium, copper and manganese (EPQ).

Cancer Cell Line and in Vivo Design	Tumor Growth and Metastasis Inhibition	In Vitro Inhibition
Athymic female mice inoculated subcutaneously with 3 × 10^6^ ovarian ES-2 cells Control group fed regular murine diet and EPQ group diet supplemented with EPQ 0.5% × 4 weeks [109]	Tumor weight reduced by 59.2% (*p* < 0.0001) and tumor burden by 59.7% *p* < 0.0001) in EPQ mice	ES-2 cell proliferation inhibited by 35% at 1000 µg/mL EPQ; MMP-2 virtual total block at 1000 µg/mL EPQ; cell migration and Matrigel invasion blocked at 500 µg/mL EPQ
Athymic female mice inoculated intraperitoneally with 2 × 10^6^ ovarian A-2780 cells Control group fed regular murine diet and EPQ group diet supplemented with EPQ 0.5% × 4 weeks [110]	Incidence of ovarian tumors reduced to 1 small tumor in EPQ group contrasted with Control group mice which all developed large ovarian tumors; tumor growth suppressed by 87% (*p* < 0.0001); lung metastasis completely suppressed in EPQ mice, but 100% present in Control mice	A-2780 cell proliferation inhibited by 80% at 1000 µg/mL EPQ; MMP-9 virtual total block at 250 µg/mL EPQ; Matrigel invasion blocked at 250 µg/mL EPQ
